# Engineering of *Escherichia coli* for direct and modulated biosynthesis of poly(3-hydroxybutyrate-*co*-3-hydroxyvalerate) copolymer using unrelated carbon sources

**DOI:** 10.1038/srep36470

**Published:** 2016-11-07

**Authors:** Kajan Srirangan, Xuejia Liu, Tam T. Tran, Trevor C. Charles, Murray Moo-Young, C. Perry Chou

**Affiliations:** 1 Department of Chemical Engineering , University of Waterloo, 200 University Avenue West Waterloo, Ontario N2L 3G1 Canada; 2 Department of Biology, University of Waterloo, Waterloo, Ontario, Canada

## Abstract

While poly(3-hydroxybutyrate-*co*-3-hydroxyvalerate) [P(3HB-*co*-3HV)] is a biodegradable commodity plastic with broad applications, its microbial synthesis is hindered by high production costs primarily associated with the supplementation of related carbon substrates (e.g. propionate or valerate). Here we report construction of engineered *Escherichia coli* strains for direct synthesis of P(3HB-*co*-3HV) from an unrelated carbon source (e.g. glucose or glycerol). First, an *E. coli* strain with an activated sleeping beauty mutase (Sbm) operon was used to generate propionyl-CoA as a precursor. Next, two acetyl-CoA moieties or acetyl-CoA and propionyl-CoA were condensed to form acetoacetyl-CoA and 3-ketovaleryl-CoA, respectively, by functional expression of β-ketothiolases from *Cupriavidus necator* (i.e. PhaA and BktB). The resulting thioester intermediates were channeled into the polyhydroxyalkanoate (PHA) biosynthetic pathway through functional expression of acetoacetyl-CoA reductase (PhaB) for thioester reduction and PHA synthase (PhaC) for subsequent polymerization. Metabolic engineering of *E. coli* host strains was further conducted to enhance total PHA content and the 3-hydroxyvaleryl (3HV) monomer fraction in the copolymer. Using a selection of engineered *E. coli* strains for batch cultivation with an unrelated carbon source, we achieved high-level P(3HB-*co*-3HV) production with the 3HV monomer fraction ranging from 3 to 19 mol%, demonstrating the potential industrial applicability of these whole-cell biocatalysts.

Since the 1970s, petroleum-derived (i.e. traditional) plastics have been deemed as the most used material in the world with a myriad of domestic, medical, and commercial applications^1^,^2^. The annual global production of polymeric materials is estimated to be approximately 200 million tons^2^. Mounting concerns associated with fossil fuels (e.g. price stability, source depletion) and environment (e.g. climate change, plastic degradability) have created a renewed impetus in developing more sustainable bio-based production platforms. As a result, natural biodegradable polymers, such as polyhydroxyalkanoates (PHAs), have attracted considerable interest as promising candidates to replace petroleum-based plastics. PHAs are polyesters with various (*R*)-hydroxycarboxylic acids as monomers and their physical and mechanical properties are highly dependent on the monomeric composition^3^,^4^. PHAs are produced by diverse Gram-negative and Gram-positive bacteria, including those from the genera *Cupriavidus, Pseudomonas*, and *Bacillus*, and exist as insoluble cytoplasmic granules as carbon storage and reducing potential^4^,^5^,^6^.

The most well-characterized and naturally abundant member of PHAs is poly(3-hydroxybutyrate) (PHB)^4^,^7^. The precursor of PHB, i.e. acetoacetyl-CoA, is a C4 biogenic intermediate formed through Claisen condensation of two acetyl-CoA moieties, which is catalyzed by β-ketothiolase (PhaA) (Fig. 1). Acetoacetyl-CoA is reduced to (*R*)-3-hydroxybutyryl-CoA (3HB-CoA) by an NADPH-dependent acetoacetyl-CoA reductase (PhaB). As the monomer, 3HB-CoA is then incorporated into the growing polymer by PHA synthase (PhaC)^6^. Similar to other short-chain PHA homopolymers, PHB has a high degree of crystallinity and a high melting temperature. Therefore, PHB has a limited range of applicability as an industrial plastic material since it is too brittle and stiff to be processed^5^,^8^. Accordingly, numerous attempts have been made to develop copolymers with modulated properties (e.g. increased toughness, ductility, and impact strength as well as lower stiffness and crystallinity) by structurally incorporating longer-chain (*R*)-hydroxycarboxylic acid monomers into PHB.

Being a coalesce of monomers 3HB-CoA and (*R*)-3-hydroxyvaleryl-CoA (3HV-CoA), poly(3-hydroxybutyrate-*co*-3-hydroxyvalerate) [P(3HB-*co*-3HV)] has been the most extensively studied 3HB-based copolymer historically. P(3HB-*co*-3HV) was commercially sold by Imperial Chemical Industries and later Monsanto under the tradename Biopol™^9^,^10^. While Monsanto had several P(3HB-*co*-3HV)-based biodegradable products in the pipeline such as molded bottles, films, coatings, and even biochemical devices, the company withdrew from the biopolymer market in 1998 citing high production costs as a major deterrent to Biopol™ commercialization^4^,^11^. A major factor for the high production costs was associated with exogenous supplementation of a related carbon source (i.e. propionate or valerate) to cultures of *Cupriavidus necator*, a natural producer of PHAs^9^,^12^,^13^. Though such carbon supplementation was deemed critical for not only supplying propionyl-CoA *in vivo* but also controlling the monomer composition of P(3HB-*co*-3HV) as *C. necator* does not produce propionyl-CoA naturally, it is prohibitively expensive and can negatively impact cell growth and culture performance^14^.

Here we report direct and propionate-independent biosynthesis of P(3HB-*co*-3HV) using engineered *Escherichia coli* strains with an unrelated carbon source. To generate the required 3HV monomer for incorporation into the polymeric chain, we first introduced a biocatalytic node toward propionyl-CoA as a precursor. Common microorganisms amenable to genetic manipulation, such as *E. coli* or yeast, often do not process metabolic pathways or enzymes for propionyl-CoA anabolism^15^. Previously, we reported genomic activation of a quiescent yet functional sleeping beauty mutase (Sbm) operon in *E. coli*^16^,^17^,^18^. This four-gene operon (i.e. *sbm-ygfD*-*ygfG*-*ygfH*) encodes various enzymes for conversion of the tricarboxylic acid (TCA) cycle intermediate succinyl-CoA to propionyl-CoA, resulting in the production of 1-propanol and propionate (Fig. 1)^19^. The propionogenic *E. coli* with an activated Sbm operon for *de novo* biosynthesis of propionyl-CoA was used as the basic production host.

For P(3HB-*co*-3HV) formation in the propionogenic *E. coli*, three more biosyntheses were implemented. First, two acetyl-CoA moieties or acetyl-CoA and propionyl-CoA were condensed to form thioester intermediates acetoacetyl-CoA and 3-ketovaleryl-CoA, respectively, by expressing β-ketothiolases from *C. necator* (i.e. PhaA and BktB). Next, by expressing acetoacetyl-CoA reductase from *C. necator* (i.e. PhaB), the thioester intermediates were reduced to two PHA monomers 3HB-CoA and 3HV-CoA, respectively. Finally, by expressing PHA synthase from *C. necator* (i.e. PhaC), the two PHA monomers were included into the growing chain of PHA for polymerization (Fig. 1). In addition to the biosynthetic approaches, metabolic engineering of *E. coli* host strains by inactivating key genes involved in the fermentative or glycerol-dissimilation pathways was conducted to enhance total PHA content and the 3HV monomer fraction in the copolymer. Using a selection of our engineered *E. coli* strains for batch cultivation with an unrelated carbon source, we achieved high-level P(3HB-*co*-3HV) production with the 3HV monomer fraction ranging from 3 to 19 mol%, which is similar to that of commercial Biopol™, thus demonstrating the potential industrial applicability of these whole-cell biocatalysts.

## Results

### Direct biosynthesis of P(3HB-co-3HV) in *E. coli*

**Figure 1:**
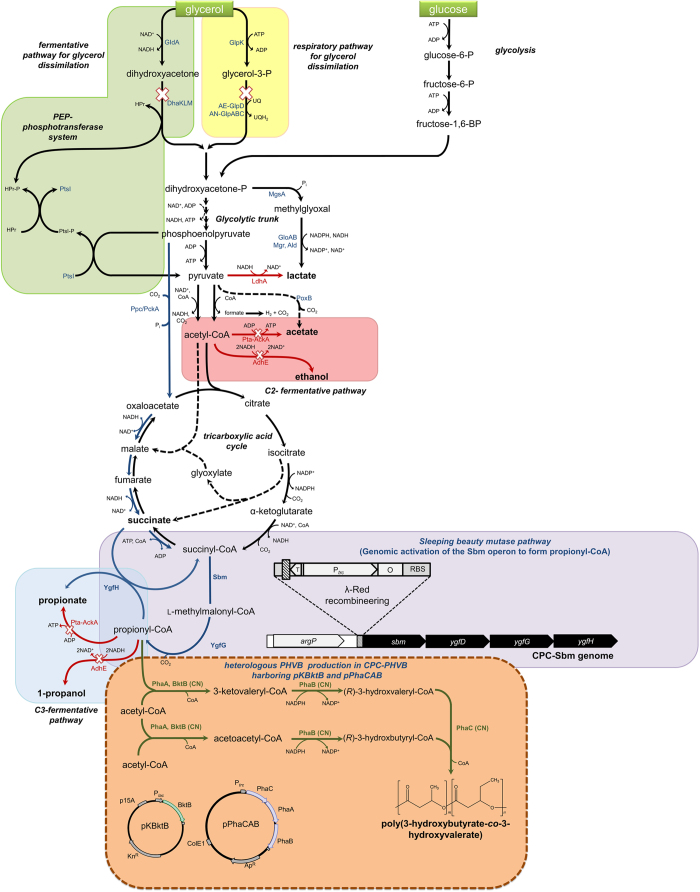
Schematic representation of the synthetic biology and metabolic strategies used to establish the P(3HB-*co*-3HV) biosynthetic pathway in engineered *E. coli*. Heterologous enzymes from Cupriavidus necator (CN) are shown in green text. The fermentative pathway for glycerol dissimilation is presented in a light green box and the respiratory pathway for glycerol dissimilation is presented in a yellow box. The activated Sleeping beauty mutase (Sbm) pathway is presented in a purple box. Red and blue arrows represent the route to the C2- and C3-fementative products, respectively. The C2-fermentative pathway is presented in a red box, while the C3-fermentative pathway is presented in a blue box. Relevant enzymes for production of various fermentative products as well as the enzymes of the respiratory and fermentative glycerol pathways and the Sbm pathway are in blue text. Disrupted metabolic pathways are indicated with a cross (X) symbol.

A prerequisite to the formation of 3HV-CoA in *E. coli* is the intracellular presence of propionyl-CoA as a precursor. Recently, we reported construction of engineered *E. coli* strains for heterologous production of 1-propanol^17^,^18^ and propionate^16^ by activating the inherently silent sleeping beauty mutase (Sbm) operon in the host genome. The Sbm pathway serves as a direct route to propionyl-CoA through extended dissimilation of succinyl-CoA, a TCA cycle intermediate (Fig. 1). In this study, the resulting propionogenic *E. coli*, CPC-Sbm, and its derivatives were explored as host strains to produce P(3HB-*co*-3HV).

To implement relevant pathways for the production of P(3HB-*co*-3HV), a double plasmid expression system was employed (Fig. 1 and Table S1). First, using a β-ketothiolase encoded by the *bktB* gene from *C. necator*, a pathway for carbon chain elongation was established to condense propionyl-CoA and acetyl-CoA or two acetyl-CoA molecules to form biogenic intermediates 3-ketovaleryl-CoA and acetoacetyl-CoA, respectively. The *bktB* gene was cloned in a plasmid pK-BktB with a p15A replicon for its heterologous expression under the regulation of the *lac* promoter. Next, for reduction of 3-ketovaleryl-CoA and acetoacetyl-CoA to their corresponding 3-hydroxyacyl-CoA monomers, i.e. 3HV-CoA and 3HB-CoA, and subsequent polymerization of them, we introduced the *C. necator* PHA biosynthetic operon in the transcriptional order *phaC*-*phaA*-*phaB* in a plasmid pPhaCAB with a ColE1 replicon for its heterologous expression under the regulation of the *trc*-promoter. Note, both BktB and PhaA have biosynthetic thiolase activity, with BktB having a greater specificity toward 3-ketovaleryl-CoA than acetoacetyl-CoA^20^. To generate the PHA-producing strains CPC-PHBV and CPC-PHB, the two plasmids pK-BktB and pPhaCAB were co-transformed into the propionogenic strain CPC-Sbm and its non- propionogenic parental strain BW-∆ldhA, respectively.

**Figure 2:**
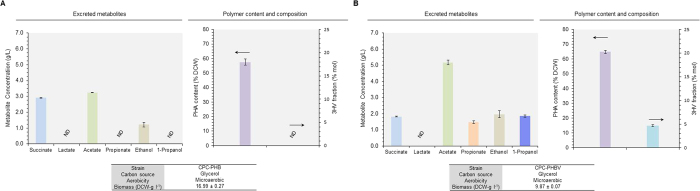
Establishing a direct biosynethic pathway for P(3HB) and P(3HB-*co*-3HV) copolymer formation in *E. coli*. Major metabolite titers, total PHA content (% DCW) and 3HV in total polymer (% mol) during microaerobic cultivations of strains (**A**) CPC-PHB and (**B**) CPC-PHBV using glycerol as the major carbon source. Culture performance (i.e. overall glycerol consumption and final biomass and metabolite concentrations) and time profiles are given in Fig. S1. The data represent the means ± S.D. (n = 3). P < 0.05.

To demonstrate P(3HB-*co*-3HV) production from an unrelated carbon source, CPC-PHBV and CPC-PHB were cultivated in a bioreactor under microaerobic conditions with 30 g l^−1^ glycerol as the sole carbon source (Fig. 2 and S1). For CPC-PHBV, the total PHA content accounted for 65% dry cell weight (DCW) with 4.7 mol% of the total PHA being 3HV. On the other hand, for the control strain CPC-PHB, the total PHA content accounted for only 57% DCW with no 3HV fraction. The results suggest competent production of P(3HB-*co*-3HV) for CPC-PHBV with a propionogenic host background, but not for CPC-PHB with a non- propionogenic host background. No PHA production was observed for the other control strains that do not harbor pPhaCAB (data not shown), indicating that the *C. necator* PHA biosynthetic operon was functionally expressed. The results also suggest that the presence of Sbm-derived propionyl-CoA can mediate P(3HB-*co*-3HV) biosynthesis from an unrelated carbon source. Upon comparing CPC-PHB and CPC-PHBV cultivations, activation of the Sbm operon ostensibly decreased both glycerol dissimilation rate and biomass yield, but with enhanced production of C2- and C3-fermentative metabolites.

### Cultivation conditions for P(3HB-*co*-3HV) biosynthesis

**Figure 3:**
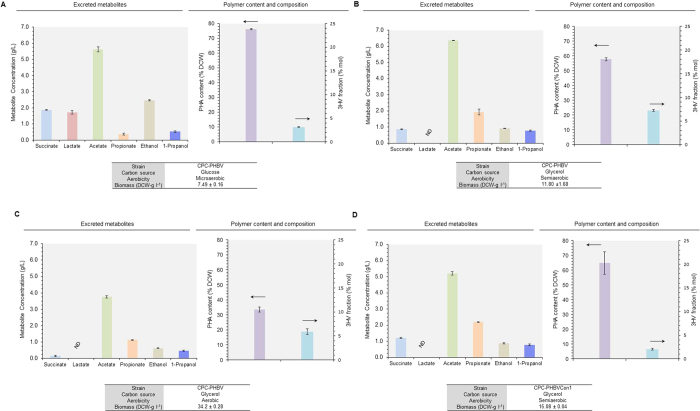
Cultivation conditions for enhancing 3HV incorporation in the copolymer. Major metabolite titers, total PHA content (% DCW) and 3HV in total polymer (% mol) during (**A**) microaerobic cultivation of CPC-PHBV using glucose as the major carbon source or (**B**) semiaerobic culitvation of CPC-PHBV using glycerol as the major carbon source. (**C**) Major metabolite titers, total PHA content (% DCW) and 3HV in total polymer (% mol) during aerobic cultivation of CPC-PHBV using glycerol as the major carbon source. (**D**) Major metabolite titers, total PHA content (% DCW) and 3HV in total polymer (% mol) during semiaerobic cultivation of CPC-PHBVCon1 using glycerol as the major carbon source. Culture performance (i.e. overall glycerol or glucose consumption and final biomass and metabolite concentrations) and time profiles are given in Fig. S2. The data represent the means ± S.D. (n = 3). P < 0.05.

With the established pathway for direct biosynthesis of P(3HB-*co*-3HV) in CPC-PHBV, the effects of bioreactor conditions (i.e. carbon source and aerobicity) on culture performance and copolymer formation were investigated (Fig. 3 and S2). While the carbon dissimilation rate for glucose culture was much faster than that for glycerol culture (i.e. taking ~54 h and ~21 h to consume 30 g l^−1^ glycerol and glucose, respectively) under microaerobic conditions, the biomass and PHA yield for glucose culture was 24% and 10%, respectively, lower than those for glycerol culture (i.e. 5.8 g-PHA l^−1^ for glucose culture vs. 6.4 g-PHA l^−1^ for glycerol culture; Fig. 2B vs. 3A). Most importantly, the 3HV fraction of the produced PHA was also reduced for glucose culture (i.e. 3.2 mol% for glucose culture vs. 4.7 mol% for glycerol culture). While the titers of the C2-fermentative metabolites (i.e. ethanol and acetate) for the two cultures remained similar, more C3-fermentative metabolites (i.e. 1-propanol and propionate) were produced for glycerol culture. Also, note that, though CPC-PHBV has a *∆ldhA* genetic background, an unusually high lactate spill was observed for glucose culture. These results corroborate with our previous observations that glycerol, with a higher reductance, appears to be a more effective carbon source than glucose to drive more carbon flux toward the C3-fermentative pathways when the genomic Sbm operon is activated^16^,^18^.

On the other hand, introducing semiaerobic conditions into the culture to make it more oxygenic significantly increased glycerol dissimilation rate (i.e. taking ~21 h and ~54 h to consume 30 g l^−1^ glycerol for semiaerobic and microaerobic cultivations, respectively) and enhanced cultivation performance. With a slightly higher PHA yield (i.e. 6.8 g-PHA l^−1^ for semiaerobic culture vs. 6.4 g-PHA l^−1^ for microaerobic culture; Fig. 2B vs. 3B), semiaerobic cultivation also dramatically increased the 3HV fraction of the produced PHA (i.e. 7.2 mol% for semiaerobic culture vs. 4.7 mol% for microaerobic culture). Note that semiaerobic cultivation resulted in a higher level of acidogenesis with the sum of acetate and propionate production accounting for up to 40% of dissimilated glycerol. Nevertheless, such operational change increased the C3/C2 fermentative metabolite ratio (i.e. 0.31 for semiaerobic culture vs. 0.11 for microaerobic culture), implying that the Sbm operon remained active under semiaerobic conditions. Further increasing the air-purging rate at 1 vvm into the bulk culture (i.e. aerobic cultivation), however, reduced PHA yield (i.e. 6.8 g-PHA l^−1^ for semiaerobic culture vs. 5.7 g-PHA l^−1^ for aerobic culture; Fig. 3B vs. 3C) with most dissimilated glycerol being directed toward biomass formation though 3HV fraction was unaffected. Based on these results, it appears that semiaerobic cultivation with glycerol as the major carbon source was most suitable for P(3HB-*co*-3HV) production and, therefore, all subsequent cultivations were conducted under this culture condition.

Note that BktB has been previously shown to have a higher substrate specificity toward C5 thioesters, compared to PhaA which is a short-chain-specific thiolase^20^,^21^. Eliminating expression of *bktb* in strain CPC-PHBVCon1 drastically reduced the 3HV fraction in the copolymer, compared with that obtained in strain CPC-PHBV (i.e. 2.0 mol% and 7.2 mol% for CPC-PHBVCon1 and CPC-PHBV, respectively) under semiaerobic conditions (Fig. 3D). Thus, simultaneous expression the two biosynthetic thiolases of PhaA and BktB was synergistically ideal for P(3HB-*co*-3HV) production.

### Metabolic engineering of fermentative pathways to enhance P(3HB-*co*-3HV) production

**Figure 4:**
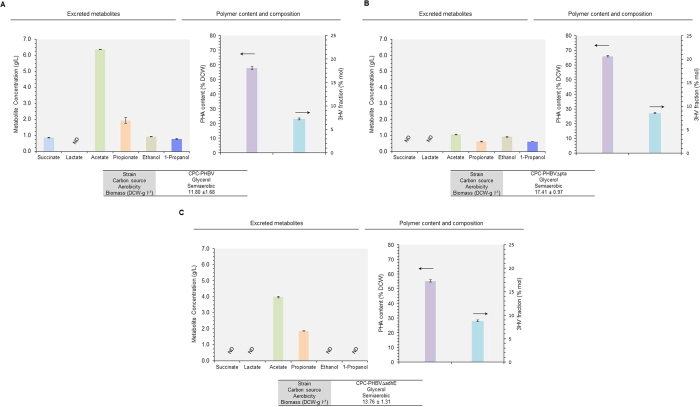
Metabolic engineering strategies enhance copolymer formation. Major metabolite titers, total PHA content (% DCW) and 3HV in total polymer (% mol) during semiaerobic cultivations of (**A**) CPC-PHBV, (**B**) CPC-PHBV∆adhE and (**C**) CPC-PHBV∆pta using glycerol as the major carbon source. Culture performance (i.e. overall glycerol consumption and final biomass and metabolite concentrations) and time profiles are given in Fig. S3. The data represent the means ± S.D. (n = 3). P < 0.05.

While P(3HB-*co*-3HV) production was markedly improved for CPC-PHBV under semiaerobic conditions, high-level acidogenesis was observed with an excessive accumulation of acetate and propionate in the culture medium. Such acidogenesis can potentially exacerbate carbon spill and reduce P(3HB-*co*-3HV) yield. Accordingly, we blocked acidogenesis by inactivating the phosphotransacetylase (Pta)-acetate kinase (AckA) pathway in CPC-PHBV and culture performance of the resultant mutant CPC-PHBV∆pta was evaluated (Fig. 4B and S3B). While the overall glycerol dissimilation rate for CPC-PHBV∆pta was slightly lower than that for CPC-PHBV (i.e. taking ~29 h and ~21 h to consume 30 g l^−1^ glycerol for CPC-PHBV∆pta and CPC-PHBV, respectively), the level of acidogenesis was significantly reduced with an abolished succinate production. In addition, the biomass yield for CPC-PHBV∆pta was 48% higher than that for CPC-PHBV with a concomitant increase in total PHA content (i.e. 66% DCW and 57% DCW for CPC-PHBV∆pta and CPC-PHBV, respectively). Most importantly, the 3HV fraction of the produced PHA was increased (i.e. 8.5 mol% and 7.2 mol% for CPC-PHBV∆pta and CPC-PHBV, respectively).

While the level of solventogenesis was not considerably high for CPC-PHBV, the carbon spill associated with alcohol formation can potentially limit P(3HB-*co*-3HV) biosynthesis. Therefore, we blocked solventogenesis in CPC-PHBV by inactivating the *adhE* gene encoding the fermentative bifunctional acetaldehyde/alcohol dehydrogenase and culture performance of the resulting mutant CPC-PHBV∆adhE was evaluated (Fig. 4C and S3C). Compared to the control strain CPC-PHBV, the overall glycerol dissimilation rate for CPC-PHBV∆adhE was slower (i.e. taking ~33 h and ~21 h to consume 30 g l^−1^ glycerol for CPC-PHBV∆adhE and CPC-PHBV, respectively), whereas the biomass yield was 17% higher. The higher biomass yield resulted in a higher PHA yield (i.e. 7.7 g-PHA l^−1^ and 6.8 g-PHA l^−1^ for CPC-PHBV∆adhE and CPC-PHBV, respectively). Most importantly, the 3HV fraction of the produced PHA was increased (i.e. 8.8 mol% and 7.2 mol% for CPC-PHBV∆adhE and CPC-PHBV, respectively). These results suggest that inactivating the genes involved in the fermentative pathways for reduced acidogenesis and/or solventogenesis can potentially lead to more effective production of P(3HB-*co*-3HV).

### Manipulation of glycerol dissimilation to enhance P(3HB-*co*-3HV) biosynthesis

**Figure 5:**
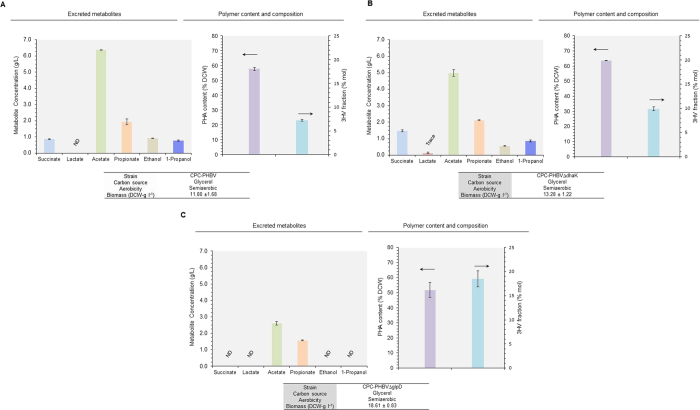
Linking glycerol metabolism to P(3HB-*co*-3HV) biosynthesis in *E. coli*. Major metabolite titers, total PHA content (% DCW) and 3HV in total polymer (% mol) during semiaerobic cultivations of (**A**) CPC-PHBV, (**B**) CPC-PHBV∆dhaKand (**C**) CPC-PHBV∆glpD using glycerol as the major carbon source. Culture performance (i.e. overall glycerol consumption and final biomass and metabolite concentrations) and time profiles are given in Fig. S4. The data represent the means ± S.D. (n = 3). P < 0.05.

In *E. coli*, glycerol is dissimilated via two routes to form the glycolytic intermediate dihydroxyacetone phosphate (DHAP), i.e. (i) the GldA-DhaK route under fermentative conditions and (ii) the GlpK-GlpD/GlpABC route under respiratory conditions^22^ (see Fig. 1). Manipulation of various genes involved in the respiratory and fermentative pathways for glycerol dissimilation appears to be an effective method to drive more carbon flux toward the propionyl-CoA node^16^. Among these trials, inactivation of either the fermentative dihydroxyacetone kinase (∆*dhaK*) or respiratory glycerol-3-phosphate dehydrogenase (∆*gldA*) pathway almost abolished solventogenesis with more carbon flux being shifted toward the C3-fermentative pathway. Accordingly, for better understanding of the effects of glycerol dissimilation on P(3HB-*co*-3HV) biosynthesis, we further engineered CPC-PHBV by inactivating either of the two pathways, resulting in the derivation of two mutant strains, i.e. CPC-PHBV∆dhaK and CPC-PHBV∆glpD containing a single deletion in *dhaK* and *glpD*, respectively, and their culture performance was evaluated (Fig. 5B–C and S4B–C).

Compared to the control strain CPC-PHBV, inactivation of the fermentative GldA-DhaK route slightly reduced glycerol dissimilation rate (i.e. taking 27 h and 21 h to consume 30 g l^−1^ glycerol for CPC-PHBV∆dhaK and CPC-PHBV, respectively) with a slight increase in both biomass yield (i.e. 66% DCW and 57% DCW for CPC-PHBV∆dhaK and CPC-PHBV, respectively) and total PHA content (i.e. 8.4 g l^−1^ and 6.8 g l^−1^ for CPC-PHBV∆dhaK and CPC-PHBV, respectively). While the inactivation minimally affected metabolite profiling, the 3HV fraction of the produced PHA was significantly increased by 36% (i.e. 9.8 mol% and 7.2 mol% for CPC-PHBV∆pta and CPC-PHBV, respectively).

On the other hand, compared to the control strain CPC-PHBV, though inactivation of the aerobic GlpK-GlpD/GlpABC route significantly hampered glycerol dissimilation (i.e. taking 41 h and 21 h to consume 30 g l^−1^ glycerol for CPC-PHBV∆glpD and CPC-PHBV, respectively), the biomass yield was significantly increased by 58%. The gene inactivation potentially reduced carbon spill by completely blocking solventogenesis and significantly reducing acidogenesis. As a result, the total amount of PHA production was significantly increased (i.e. 9.6 g l^−1^ and 6.8 g l^−1^ for CPC-PHBV∆glpD and CPC-PHBV, respectively). Most importantly, the 3HV fraction reached to an extremely high level of 18.5 mol%, which, to the best of our knowledge, represents the highest reported level for *E. coli*-based P(3HB-*co*−3HV) biosynthesis using an unrelated carbon source.

## Discussion

While biological synthesis based on whole-cell biocatalysts is an attractive renewable approach for manufacturing, it is often limited by the availability of natural pathways to form molecules of interest. Economic microbial production of P(3HB-*co*-3HV) has been limited by high production costs associated with supplementation of related carbons, such as propionate and valerate, due to the lack of intracellular propionyl-CoA as a precursor in the biotransformation systems. We recently conducted heterologous production of 1-propanol and propionate via a C3-fermentative pathway with propionyl-CoA as a hub through genomic activation of the Sbm operon in *E. coli*^16^,^17^,^18^. Establishing the metabolite node of propionyl-CoA in *E. coli* opens an avenue for microbial synthesis of a selection of non-native chemicals from unrelated carbon sources. On the other hand, implementing the CoA-dependent Claisen condensation pathway in *E. coli* enables carbon chain elongation for novel biosynthesis^23^,^24^,^25^. In this report, we further engineered our propionogenic *E. coli* by expressing β-ketothiolases for CoA-dependent Claisen condensation of two acetyl-CoA moieties or acetyl-CoA and Sbm-derived propionyl-CoA. The resulting C4 and C5 biogenic thioesters (i.e. acetoacetyl-CoA and 3-ketovaleryl-CoA, respectively) were then channeled into the PHA synthesis pathway for subsequent reduction and polymerization. Note that the two β-ketothiolases of PhaA and BktB can synergistically enhance molecular fusion of two CoA-moieties and, therefore, the polymer-producing capacity of the strain. These metabolic and genetic engineering strategies led to the development of a selection of engineered *E. coli* strains capable of direct biosynthesis of P(3HB-*co*-3HV) with various 3HV fractions in the copolymer from a single unrelated carbon source (i.e. glucose or glycerol).

Several attempts for P(3HB-*co*-3HV) production from unrelated carbon sources in natural and recombinant microbes^14^,^26^,^27^,^28^,^29^ were made. These approaches generally rely on the extended dissimilation of either citramalate or l-threonine through the 2-keto-acid biosynthesis pathways to form propionyl-CoA. For a comprehensive review of P(3HB-*co*-3HV) production in natural and non-natural hosts, see^30^.While the 2-keto-acid biosynthesis pathways are extensively utilized for the production of biofuels^31^,^32^ and organic acids^33^, their application for direct P(3HB-*co*-3HV) synthesis is limited as the 3HV fractions of the copolymer obtained from these strategies are often below the sufficient levels for industrial adoption, indicating potential intracellular shortage of the propionyl-CoA precursor. For instance, engineering *E. coli* to synthesize P(3HB-*co*-3HV) from propionyl-CoA derived from citramalate resulted in a 3HV fraction of ~5 mol%^13^. While combining the citramalate and l-threonine pathways to form the propionyl-CoA precursor markedly increased the 3HV fraction of P(3HB-*co*-3HV) up to 25 mol%, the total amount of PHA production of 2–14% DCW was relatively low^26^. For extending the carbon chain, accumulation of key biogenic precursors of acetyl-CoA and propionyl-CoA is critical to overcome the high thermodynamic barrier of the condensation reactions catalyzed by the β-ketothiolases^34^. Based on the current results, the Sbm pathway appears to be far more conducive in accumulating propionyl-CoA as relatively high levels of 3HV fractions of the copolymer were produced using unrelated carbon sources. This may in part be explained by the fact that the Sbm pathway serves as a direct route toward propionyl-CoA from the glycolytic intermediate phosphoenolpyruvate (PEP), making it potentially more efficient than the 2-keto-acid-based pathways. The Sbm pathway is also devoid of amino acid biosynthetic intermediates and therefore not subjected to complex feedback regulations. Rerouting carbon flux via amino acid intermediates toward propionyl-CoA can potentially perturb amino acid metabolism and result in growth retardation and low protein production^14^,^35^.

The physiochemical and mechanical properties of P(3HB-*co*-3HV) are highly contingent on the relevant monomer composition of the copolymer^29^,^36^. Accordingly, in addition to the total copolymer amount of the culture, it is often crucial to control the 3HV fraction of P(3HB-*co*-3HV) suitable for a wide spectrum of applications. While the 3HV fraction can be easily fine-tuned by adjusting the concentration and/or feeding rate of the related carbons for those bioprocesses with exogenous supplementation of related carbons^37^, such modulation in intracellular copolymer composition is often difficult when P(3HB-*co*-3HV) is made from unrelated carbons. Previously, a “dial-a-composition” system was proposed whereby the copolymer composition was altered and to some extent controlled at a fixed carbon concentration in the culture by varying the level of induction of critical pathway genes in polymer-accumulating recombinant *Salmonella*^29^,^38^. While P(3HB-*co*-3HV) was produced directly from an unrelated carbon with the control strain CPC-PHBV, the HV fraction of the copolymer was relatively low. Further metabolic engineering strategies based on inactivation of key host genes involved in either the fermentative or glycerol dissimilation pathways could potentially direct more dissimilated carbon flux toward the C3-fermentative pathway, increasing the intracellular availability of the propionyl-CoA precursor and, as a result, the HV fraction of the copolymer. The collection of various metabolically engineered *E. coli* strains, including CPC-PHBV∆adhE, CPC-PHBV∆pta, and CPC-PHBV∆glpD, serves as the platform for microbial production of P(3HB-*co*-3HV) with a wide range of HV fraction (3–19 mol%), which is similar to the diversified specifications of commercial Biopol™ for different applications. Such metabolic approach for modulating the copolymer composition appears to be more robust, consistent, and reproducible.

Our results suggest that culture performance for P(3HB-*co*-3HV) production, in terms of biomass formation, total copolymer content, and 3-HV fraction, was highly dependent on the oxygenic level of the culture. This can be due to the fact that various intracellular reactions associated with P(3HB-*co*-3HV) biosynthesis have different oxygenic preferences. For example, while polymerization is growth-associated and therefore can be favored by aerobiosis, formation of the propionyl-CoA precursor requires an anaerobic condition^39^,^40^,^41^,^42^. Nevertheless, anaerobic conditions can exacerbate carbon spill as fermentative metabolites, negatively affecting P(3HB-*co*-3HV) biosynthesis. Also, genomic activation of the Sbm operon introduces an intracellular competition in carbon flux between the C2-fermentative pathway (with acetyl-CoA as a local hub) and the C3-fermentative one (with propionyl-CoA as a local hub). While using glycerol as the major carbon source tends to drive more carbon flux toward the C3-fermentative pathway^16^, glycerol is considered as a recalcitrant feedstock for *E. coli* cultivation under limited oxygenic conditions^18^. Our results suggest that incorporation of 3HV into the copolymer chain was favorable under semiaerobic conditions. Accordingly, semiaerobic conditions with low oxygenic levels were used to balance cell growth with P(3HB-*co*-3HV) biosynthesis while minimizing the formation of fermentative metabolites.

Compared to the control strain CPC-PHBV, knocking out both the *adhE* and *pta* genes significantly improved biomass and PHA yields, reduced fermentative metabolite formation, and, slightly increased the 3HV fraction. In particular, both acidogenesis and alcohologenesis were almost abolished by knocking out the *pta* gene, significantly reducing metabolite formation as carbon spill and therefore enhancing biomass and P(3HB-*co*-3HV) formation, though the glycerol dissimilation rate was slightly reduced. The slow glycerol dissimilation rate and therefore cell growth rate can be associated with decreased carbon flux towards acetyl phosphate, an intermediate in the Pta-AckA pathway that serves as an important regulator in *E. coli* for global gene expression and metabolism^43^. Excreted acetate and propionate serve as carbon sources for precursors for acetyl-CoA and propionyl-CoA, and therefore can also be converted via parallel assimilating pathways into the cell to minimize carbon loss and redox imbalance. In most examples of pathway design for recycling of acetyl-CoA, acetate consumption is enhanced either by episomally expressing the ATP-dependent Acs (acetyl-CoA synthetase) pathway^44^ or AldB–MhpF (acetaldehyde dehydrogenases)^45^.

It was previously reported that manipulation of glycerol dissimilation can potentially direct carbon flux toward the C3-fermentative pathway, leading to more formation in propionyl-CoA, under limited oxygenic conditions^16^,^46^. Note that quinone serves as an electron acceptor during aerobic glycerol metabolism, whereas glycerol is oxidized into dihydroxyacetone using NAD^+^ as the electron acceptor in the absence of oxygen (Fig. 1)^47^. For the production of reduced metabolites, cells preferentially utilize the fermentative GldA-DhaK route, through which high energy NADH is generated^48^,^49^. Indeed, metabolic manipulation to direct glycerol dissimilation through the fermentative GldA-DhaK pathway by inactivating *glpD* significantly enhanced both biomass formation and incorporation of 3HV monomer into the copolymer. Such manipulation concomitantly reduced acidogenesis and abolished alcohologenesis, though the results appear to contrast an earlier observation in which disruption of the *glpK* or *glpD* prevented cell growth under respiro-fermentative conditions^22^. Nevertheless, these results highlight the importance of the fermentative GldA-DhaK pathway for enhancing 3HV formation and its incorporation into the copolymer. Alternatively, channeling glycerol metabolism through the respiratory GlpK-GlpD/GlpABC arm by inactivating *dhaK* moderately increases the 3HV fraction in the copolymer though the fermentation appears to lean toward acidogenesis. Nonetheless, CPC-PHBV∆dhaK produced higher levels of C3 metabolites (i.e. propionate and 1-propanol) compared to CPC-PHBV. The fermentative GldA-DhaK pathway requires PEP as a cofactor and therefore serves as an important mediator in the interconversion of PEP and pyruvate^22^,^47^. Eliminating such need for PEP in CPC-PHBV∆dhaK potentially increases the intracellular PEP pool, diverting more carbon flux toward propionyl-CoA via oxaloacetate (OAA) through the reductive TCA arm. Accordingly, an effective way to harness glycerol dissimilation for generating higher levels of propionyl-CoA can be overexpression of phosphoenolpyruvate carboxylase (encoded by *ppc*) to create an efficient glycolytic node for enhanced conversion of PEP to OAA^50^,^51^.

## Methods

### Bacterial strains and plasmids

*E. coli* strains, plasmids and DNA primers used in this study are listed in Table S1. Standard recombinant DNA technologies for molecular cloning were applied^52^. *Pfu* and *Taq* DNA polymerases, T4 DNA ligase, and large (Klenow) fragment of DNA Polymerase I were obtained from New England Biolabs (Ipswich, MA, USA). All synthesized oligonucleotides were obtained from Integrated DNA Technologies (Coralville, IA, USA). DNA sequencing was conducted by the Centre for Applied Genomics at the Hospital for Sick Children (Toronto, Canada). *E. coli* BW25141 was the parental strain for derivation of all mutant strains in this study and *E. coli* HST08 was used for molecular cloning.

Activation of the genomic Sbm operon to form propionogenic *E. coli* CPC-Sbm was described previously^18^. Briefly, the FRT-Cm^R^-FRT cassette from pKD3 was PCR-amplified using the primer set c-frt, whereas the *trc* promoter-operator region was PCR-amplified using the c-ptrc primer set. The two DNA amplicons were fused together by splice overlap-extension (SOE) PCR^53^ using the forward primer of the c-frt primer set and the reverse primer of the c-ptrc primer set to generate the FRT-Cm^R^-FRT-P_*trc*_ cassette. To generate the DNA cartridge for genomic integration, the FRT-Cm^R^-FRT-P_*trc*_ cassette was PCR-amplified using the r-frt:ptrc primer set containing the 5′- and 3′-36-bp homology arms, respectively. The homology arms were chosen so that the FRT-CmR-FRT-P_*trc*_ cassette was inserted precisely upstream of the Sbm operon. λ-Red genomic recombineering was carried out as described by Datsenko and Wanner^54^.

Gene knockouts (i.e. *adhE, pta, glpD*, and *dhaK*) were introduced into CPC-Sbm by P1 phage transduction^52^ using the appropriate Keio Collection strains (The Coli Genetic Stock Center, Yale University, New Haven, CT, USA) as donors^55^. To eliminate the co-transduced FRT-Km^R^-FRT cassette, the transductant mutants were transformed with pCP20^56^, a temperature sensitive plasmid expressing a flippase (Flp) recombinase. Upon Flp-mediated excision of the Km^R^ cassette, a single Flp recognition site (FRT “scar site”) was left behind. Plasmid pCP20 was then removed by growing cells at 42 °C. The genotypes of derived knockout strains were confirmed by whole-cell colony PCR using the appropriate “verification” primer sets listed in Table S1.

The DNA fragment containing the native *C. necator* PHA operon genes (*phaC*-*phaA*-*phaB*) was PCR-amplified from the genomic DNA of the wild-type *C. necator* strain (ATCC 17699) using the g-phaCAB primer set. The amplified DNA fragment was assembled using Gibson method^57^ into vector pTrc99a. A clone with the correct transcriptional orientation of the *phaC*-*phaA*-*phaB* fragment with respect to the p_*trc*_ promoter was selected and verified by DNA sequencing, yielding pK-PhaCAB. Similarly, the β-ketothiolases gene was PCR-amplified from the genomic DNA of the wild-type *C. necator* strain (ATCC 17699) using primer set c-bktb. The resulting fragment was cloned into the *EcoR*I restriction site of pK184. A clone with the correct transcriptional orientation of the *bktB* fragment with respect to the P_*lac*_ promoter was selected and verified by DNA sequencing, yielding pKBktB.

### Media and cultivation conditions

All media components were obtained from Sigma-Aldrich Co. (St Louis, MO, USA) except glucose, yeast extract, and tryptone which were obtained from BD Diagnostic Systems (Franklin Lakes, NJ, USA). Media was supplemented with antibiotics as required (30 *μ*g mL^−1^ kanamycin and 12 *μ*g mL^−1^chloramphenicol). For PHA production, the recombinant *E. coli* strains (stored as glycerol stocks at −80 °C) were streaked on LB agar plates with appropriate antibiotics and incubated at 37 °C for 16 h. Single colonies were picked from LB plates to inoculate 30-mL SB medium (32 g l^−1^ tryptone, 20 g l^−1^ yeast extract, and 5 g l^−1^ NaCl) with appropriate antibiotics in 125-mL conical flasks. Overnight cultures were shaken at 37 °C and 280 rpm in a rotary shaker (New Brunswick Scientific, NJ, USA) and used as seed cultures to inoculate 200 mL SB media at 1% (v/v) with appropriate antibiotics in 1-L conical flasks. This second seed culture was shaken at 37 °C and 280 rpm for approximately 16 h. Cells were then harvested by centrifugation at 6,000 × g and 20 °C for 15 min and resuspended in 100-mL fresh LB media. The suspended culture was used to inoculate a 1-L stirred-tank bioreactor (CelliGen 115, Eppendorf AG, Hamburg, Germany) operated microaerobically, semiaerobically, or aerobically at 30 °C and 430 rpm. The semi-defined production medium in the bioreactor contained 30 g l^−1^ glycerol or 30 g l^−1^ glucose, 0.23 g l^−1^ K_2_HPO_4_, 0.51 g l^−1^ NH_4_Cl, 49.8 mg l^−1^ MgCl_2_, 48.1 mg l^−1^ K_2_SO_4_, 1.52 mg l^−1^ FeSO_4_, 0.055 mg l^−1^ CaCl_2_, 2.93 g l^−1^ NaCl, 0.72 g l^−1^ tricine, 10 g l^−1^ yeast extract, 10 mM NaHCO_3_, 0.2 *μ*M cyanocobalamin (vitamin B_12_) and 1,000^th^ dilution (i.e. 1 ml l^−1^) trace elements (2.86 g l^−1^ H_3_BO_3_, 1.81 g l^−1^ MnCl_2_•4H_2_O, 0.222 g l^−1^ ZnSO_4_•7H_2_O, 0.39 g l^−1^ Na_2_MoO_4_•2H_2_O, 79 *μ*g l^−1^ CuSO_4_•5H_2_O, 49.4 *μ*g l^−1^ Co(NO_3_)_2_•6H_2_O)^58^, appropriate antibiotics, and supplemented with 0.1 mM isopropyl β-D-1-thiogalactopyranoside (IPTG). Microaerobic conditions were maintained by purging air into the headspace at 0.1 vvm. Semiaerobic conditions were maintained by purging air into the bulk culture at 0.1 vvm. Aerobic conditions were maintained by purging air into the bulk culture at 1 vvm. The pH of the production culture was maintained at 7.0 ± 0.1 with 30% (v/v) NH_4_OH and 15% (v/v) H_3_PO_4_. All cultivation experiments were performed in triplicate.

### Offline analyses and polymer extraction

Culture samples were appropriately diluted with saline for measuring the optical cell density (OD_600_) using a spectrophotometer (DU520, Beckman Coulter, Fullerton, CA). Cell-free supernatant was collected and filter sterilized for titer analysis of glucose, glycerol, and the various excreted metabolites using an HPLC (LC-10AT, Shimadzu, Kyoto, Japan) with a refractive index detector (RID-10A, Shimadzu, Kyoto, Japan) and a chromatographic column (Aminex HPX-87H, Bio-Rad Laboratories, CA, USA). The column temperature was maintained at 65 °C and the mobile phase was 5 mM H_2_SO_4_ (pH 2.0) running at 0.6 mL min^−1^. The RID signal was acquired and processed by a data processing unit (Clarity Lite, DataApex, Prague, The Czech Republic).

Intracellular polymer production was evaluated by gas chromatography as described by Braunegg^59^. Briefly, culture samples harvested from the bioreactor cultivations were pelleted by centrifugation at 4000 × g for 20 min, then washed twice with distilled water, and finally dried at 100 °C overnight. The dried cell weight (DCW) was recorded before methanolysis in 2 ml chloroform and 1 ml PHA solution containing 4 g l^−1^ benzoic acid (as an internal standard) and 15% sulfuric acid in methanol. Methanolysis was carried out at 96 °C for 6 h. The reaction was then cooled to room temperature, and after addition of 1 ml distilled water, the mixture was vortexed and allowed to separate into two phases. 1 μl of the chloroform phase was injected into Agilent 6890 series GC system (Agilent Technologies, Santa Clara, CA, USA) with a J & W Scientifics DB Wax column (30 m  × 0.53 mm, film thickness 1 μM) (Agilent Technologies, Santa Clara, CA, USA). The oven program was set as following: initial temperature was set at 80 °C for 5 min, then ramped to 230 °C at 7.5 °C/min, and continued to ramp to 260 °C at a faster rate 10 °C/min followed by maintaining that temperature for the analysis. Pure standards of methyl 3-hydroxybutyrate and methyl 3-hydroxyvalerate (Sigma-Aldrich Co., St Louis, MO, USA) were used to generate calibration curves for the methanolysis assay. The PHA content was defined as the ratio of PHA mass to dry cell mass (DCW) in a given sample, expressed as a percentage. The 3HV fraction was defined as the ratio of 3HV to 3HV plus 3HB in the copolymer, expressed in mole percent.

## Additional Information

**How to cite this article**: Srirangan, K. *et al*. Engineering of *Escherichia coli* for direct and modulated biosynthesis of poly(3-hydroxybutyrate-*co*-3-hydroxyvalerate) copolymer using unrelated carbon sources. *Sci. Rep.* **6**, 36470; doi: 10.1038/srep36470 (2016).

**Publisher’s note**: Springer Nature remains neutral with regard to jurisdictional claims in published maps and institutional affiliations.

## Supplementary Material

Supplementary Information
