# Supplementary material for: Engineering of Escherichia coli for direct and modulated biosynthesis of poly(3-hydroxybutyrate-co-3-hydroxyvalerate) copolymer using unrelated carbon sources
Source: Sci Rep. 2016 Nov 7;6:36470. doi: 10.1038/srep36470 (PMC5098226; doi:10.1038/srep36470)
Supplement: Supplementary Information [file srep36470-s1.pdf]

Supplementary Information

Title: Engineering of *Escherichia coli* for direct and modulated biosynthesis of poly(3-hydroxybutyrate-*co*-3-hydroxyvalerate) copolymer using unrelated carbon sources

Authors: Kajan Srirangan, Xuejia Liu, Tam T. Tran, Trevor C. Charles, Murray Moo-Young, C. Perry Chou*

*To whom correspondence may be addressed: Email: cpchou@uwaterloo.ca

Supplementary Table Legends

1. List of *E. coli* strains and plasmids used in this study.

Supplementary Table 1

Name	Description, relevant genotype or primer sequence (5' →3')	Reference
***E. coli* host strains**		
HST08	F⁻, *endA1*, *supE44*, *thi-1*, *recA1*, *relA1*, *gyrA96*, *phoA*, *Φ80d lacZΔ M15*, Δ(*lacZYA – argF*) *U169*, Δ(*mrr – hsdRMS – mcrBC*), *ΔmcrA*, λ⁻	Takara Bio, Shiga, Japan
BW25141	F⁻, Δ(*araD-araB*)567, Δ*lacZ4787*(::*rrnB-3*), Δ(*phoB-phoR*)*580*, *λ-*, *galU95*, Δ*uidA3*::pir+, *recA1*, *endA9*(del-ins)::FRT, *rph-1*, Δ(*rhaD-rhaB*)*568*, *hsdR514*	[1]
BW25113	F⁻, Δ(*araD-araB*)*567*, Δ*lacZ4787*(::*rrnB-3*), *λ*⁻, *rph-1*, Δ(*rhaD-rhaB*)*568*, *hsdR514*	[1]
BW-ΔldhA	BW25113Δ*ldhA* null mutant	[2]
CPC-Sbm-Cm^R	BW-Δ*ldhA*, P_{trc}:*sbm* (i.e. with the FRT-Cm^R-FRT-P_{trc} cassette replacing the 204-bp upstream of the Sbm operon)	This study
CPC-Sbm	BW-Δ*ldhA*, P_{trc}:*sbm* (i.e. with the FRT -P_{trc} cassette replacing the 204-bp upstream of the Sbm operon)	This study
CPC-SbmΔadhE	BW-Δ*ldhA*, Δ*adhE*, P_{trc}:*sbm* (i.e. with the FRT -P_{trc} cassette replacing the 204-bp upstream of the Sbm operon),	This study
CPC-SbmΔpta	BW-Δ*ldhA*, Δ*pta*, P_{trc}:*sbm* (i.e. with the FRT -P_{trc} cassette replacing the 204-bp upstream of the Sbm operon)	This study
CPC-SbmΔglpD	BW-Δ*ldhA*, Δ*glpD*, P_{trc}:*sbm* (i.e. with the FRT -P_{trc} cassette replacing the 204-bp upstream of the Sbm operon)	This study
CPC-SbmΔdhaK	BW-Δ*ldhA*, Δ*dhaK*, P_{trc}:*sbm* (i.e. with the FRT- P_{trc} cassette replacing the 204-bp upstream of the Sbm operon)	This study
CPC-PHB	BW-Δ*ldhA*/pPhaCAB and pKBktB	The study
CPC-PHBV	CPC-Sbm/pPhaCAB and pKBktB	This study

CPC-PHBVCon1	CPC-Sbm/pPhaCAB	This study
CPC-PHBVΔadhE	CPC-SbmΔ*adhE*/pPhaCAB and pKBktB	This study
CPC-PHBVΔpta	CPC-SbmΔ*pta*/pPhaCAB and pKBktB	This study
CPC-PHBVΔglpD	CPC-SbmΔ*glpD*/pPhaCAB and pKBktB	This study
CPC-PHBVΔdhaK	CPC-SbmΔ*dhaK*/pPhaCAB and pKBktB	This study
**Plasmids**		
pCP20	FLP^+, λ $cI857^+$, λ p_R Rep(pSC101 ori)^{ts}, Ap^R,Cm^R	[3]
pKD46	RepA101^{ts} ori, Ap^R, *araC*-P_{araB}:*gam-bet-exo*	[1]
pTrc99a	ColE1 ori, Ap^R, P_{trc}	[4]
pKD3	R6K-γ ori, Ap^R, FRT-Cm^R-FRT	[1]
pK184	p15A ori, Km^R, P_{lac}:*lacZ'*	[5]
pPhaCAB	From pTrc99a, P_{trc}:*phaCAB*	This study
pKBktB	From pK184, P_{lac}:*bktB*	This study
**Primers**		
v-ldhA	GATAACGGAGATCGGGAATGATTAA; GGTTTAAAAGCGTCGATGTCCAGTA	[2]

v-adhE	ATCAGGTGTCCTGAACTGTGCG; TTGACCAGCGCAAATAACCCGATGA	This study
v-pta	GGCATGAGCGTTGACGCAATCA; CAGCTGTACGCGGTGATACTCAGG	This study
v-dhaK	CATCGAGGATAAACAGCGCA; ATCTGATAAAGCTCTTCCAGTGT	This study
v-glpD	CGTCAATGCTATAGACCACATC; TATTATTGAAGTTTGTAATATCCTTATCAC	This study
c-frt	AGATTGCAGCATTACACGTCTTGAG;<br>CCAGCTGCATTAATGAATCGGGCCATGGTCCATATGAATATCCTCC	This study
c-ptrc	CCGATTCATTAATGCAGCTGG; GGTCTGTTTCCTGTGTGAAATTGTTA	This study
r-frt:ptrc	**CTCGATTATGGTCACAAAGTCCTTCGTCAGGATTAA**AGATTGCAGCATTACACGTCTTGA;<br>**GTTGGCAAGCTGTTGCCACTCCTGCACGTTAGACAT**GGTCTGTTTCCTGTGTGAAATTGT	This study
v-frt:ptrc	GCGCTCGACTATCTGTTCGTCAGCTC; TCGACAGTTTTCTCCCGACGGCTCA	This study
g-phaCAB	**CACACAGGAAACAGAC**ATGGCGACCGGCAAAGGC;<br>**CGAGCTCGAATTCCAT**TCAGCCCATATGCAGGCC	This study
c-bktb	CATGATTACGAATTCGATGACGCGTGAAGTGGTAGTGGTGA;<br>TACCGAGCTCGAATTCTCAGATGCGTTCGAAGATAGCGGCAA	This study

Notation for primers: **v**- verification primer, **c**- cloning primer, **r**- recombineering and **g**-Gibson DNA assembly primer. Restriction recognition sequences are underlined and homology arms for *in vivo* or *in vitro* recombination are in bold print

Supplementary Figure Legends

1. Time profiles of glycerol, biomass, and major metabolites during (**A**) microaerobic batch cultivation of CPC-PHB and (**B**) CPC-PHBV using glycerol as the major carbon source. All of the strains were induced at the start of the batch cultivation with 0.1 mM IPTG.

2. (**A**) Time profiles of glucose, biomass, and major metabolites during microaerobic batch cultivation of CPC-PHBV using glucose as the major carbon source. Time profiles of glycerol, biomass, and major metabolites during (**B**) semiaerobic and (**C**) aerobic batch cultivation of CPC-PHBV. (**D**) Time profiles of glycerol, biomass, and major metabolites during semiaerobic batch cultivation of CPC-PHBVCon1 using glycerol as the major carbon source. All of the strains were induced at the start of the batch cultivation with 0.1 mM IPTG.

3. Time profiles of glycerol, biomass, and major metabolites during semiaerobic batch cultivation of (**A**) CPC-PHBV, (**B**) CPC-PHBVΔadhE and (**C**) CPC-PHBVΔpta using glycerol as the major carbon source. All of the strains were induced at the start of the batch cultivation with 0.1 mM IPTG.

4. Time profiles of glycerol, biomass, and major metabolites during semiaerobic batch cultivation of (**A**) CPC-PHBV, (**B**) CPC-PHBVΔdhaKand (**C**) CPC-PHBVΔglpD using glycerol as the major carbon source. All of the strains were induced at the start of the batch cultivation with 0.1 mM IPTG.

Supplementary Figure 1

A

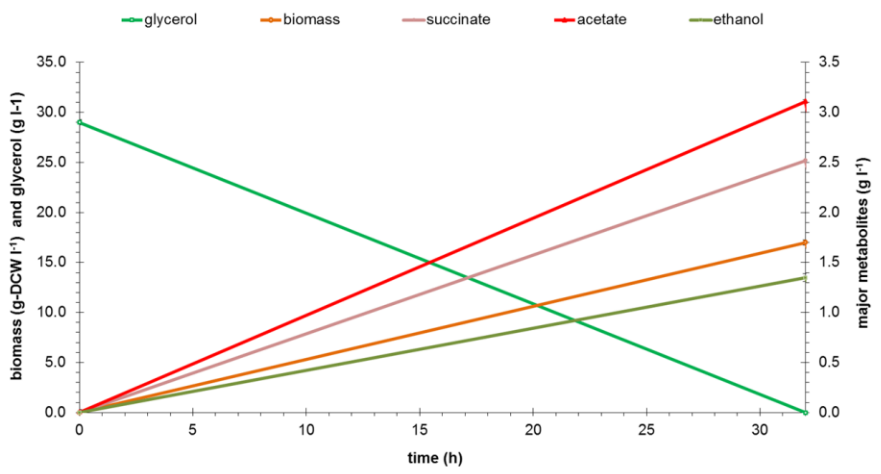

Strain	CPC-PHB
Carbon source	Glycerol
Aerobicity	Microaerobic

B

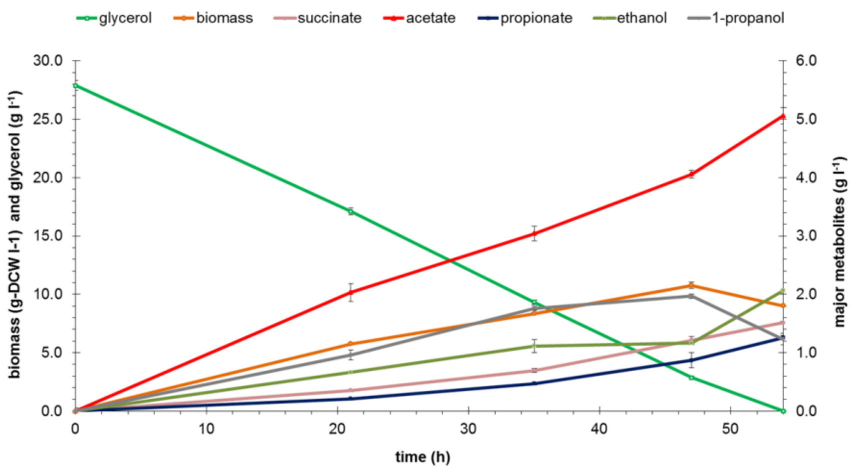

Strain	CPC-PHBV
Carbon source	Glycerol
Aerobicity	Microaerobic

Supplementary Figure 2

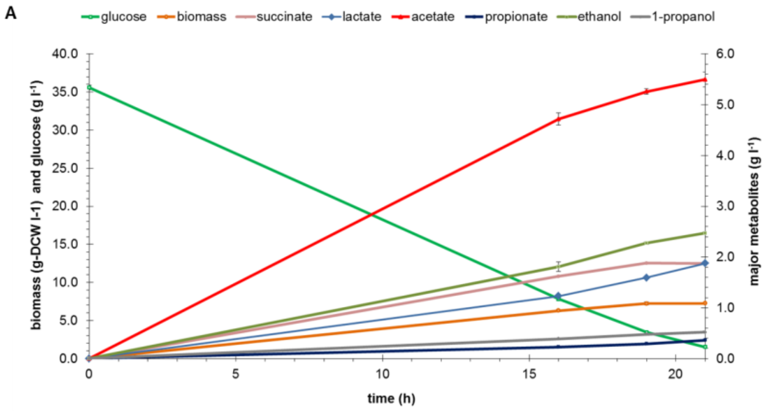

Strain	CPC-PHBV
Carbon source	Glucose
Aerobicity	Microaerobic

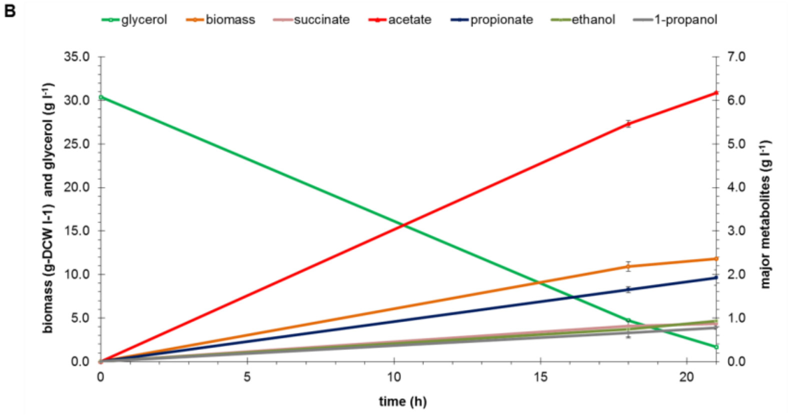

Strain	CPC-PHBV
Carbon source	Glycerol
Aerobicity	Semiaerobic

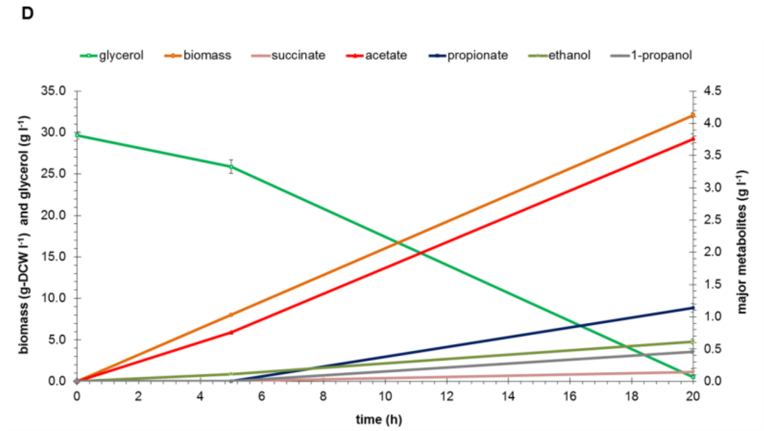

Strain	CPC-PHBV
Carbon source	Glycerol
Aerobicity	Aerobic

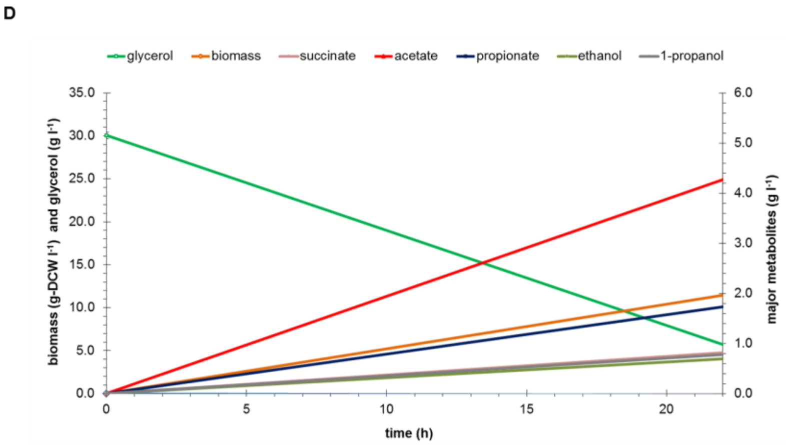

Strain	CPC-PHBVCon1
Carbon source	Glycerol
Aerobicity	Semiaerobic

Supplementary Figure 3

A

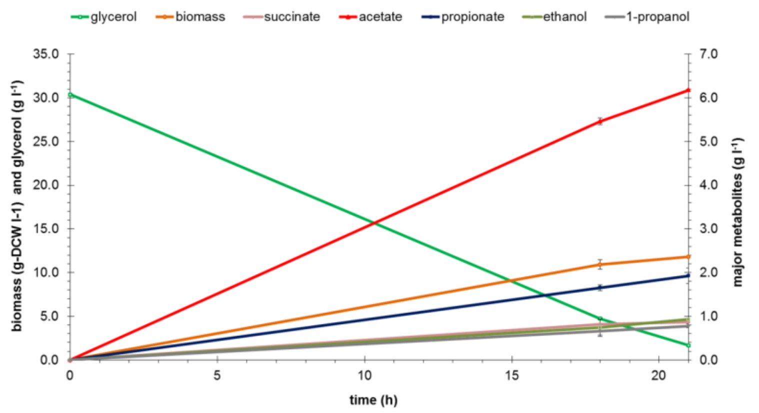

Strain	CPC-PHBV
Carbon source	Glycerol
Aerobicity	Semiaerobic

B

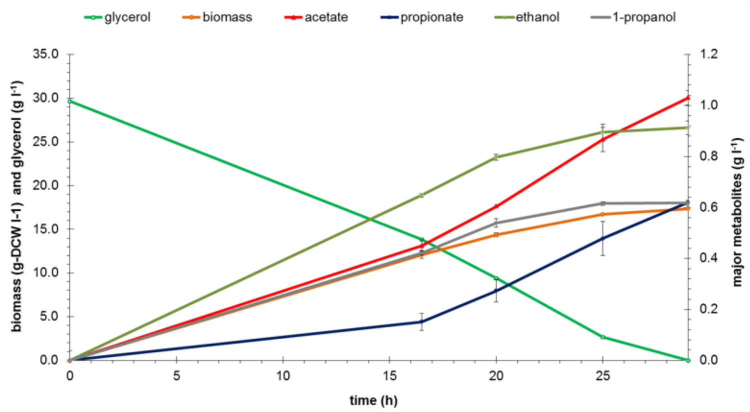

Strain	CPC-PHBVΔpta
Carbon source	Glycerol
Aerobicity	Semiaerobic

C

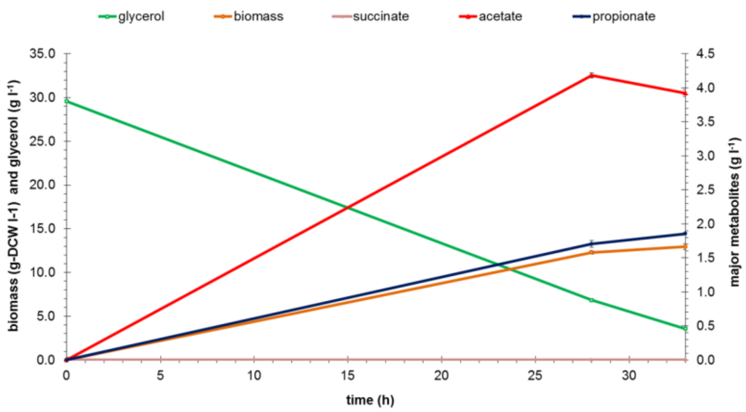

Strain	CPC-PHBVΔadhE
Carbon source	Glycerol
Aerobicity	Semiaerobic

Supplementary Figure 4

A

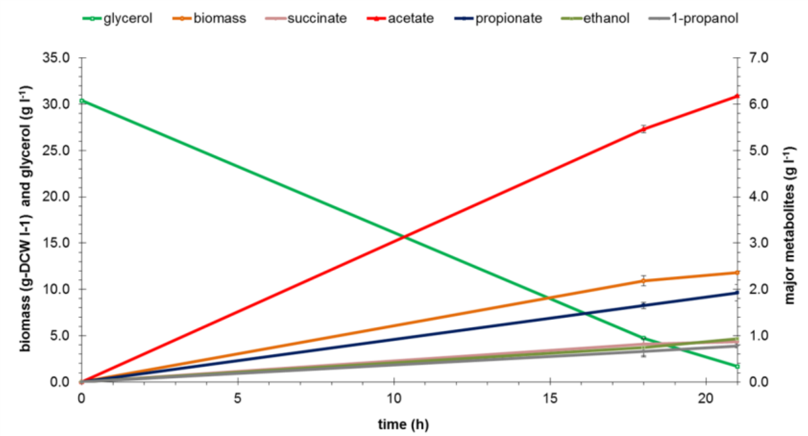

Strain<br>Carbon source<br>Aerobicity	CPC-PHBV<br>Glycerol<br>Semiaerobic

B

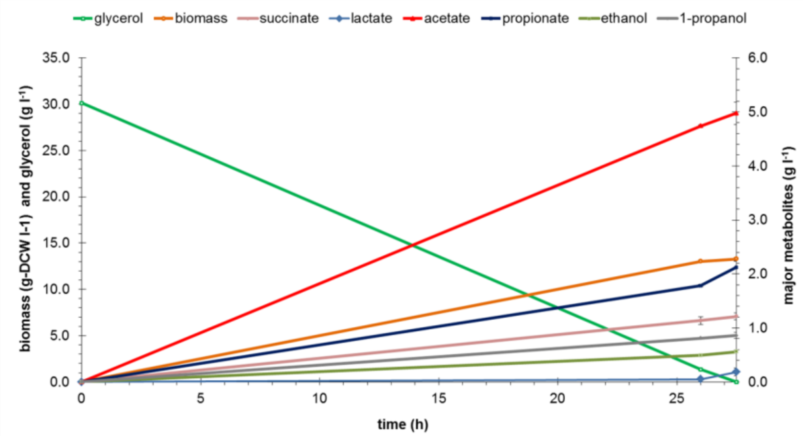

Strain<br>Carbon source<br>Aerobicity	CPC-PHBVΔdhaK<br>Glycerol<br>Semiaerobic

C

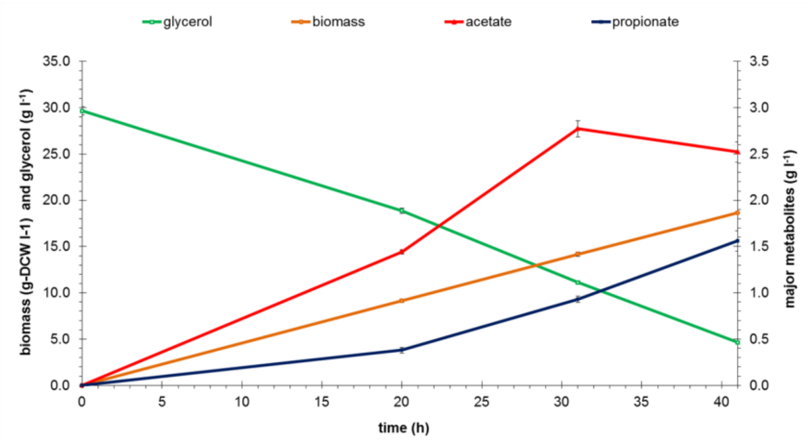

Strain<br>Carbon source<br>Aerobicity	CPC-PHBVΔglpD<br>Glycerol<br>Semiaerobic

References

1 Datsenko, K. A. & Wanner, B. L. One-step inactivation of chromosomal genes in *Escherichia coli* K-12 using PCR products. *Proc. Natl. Acad. Sci. U. S. A.* **97**, 6640-6645, (2000).

2 Srirangan, K. *et al.* Manipulating the sleeping beauty mutase operon for the production of 1-propanol in engineered *Escherichia coli*. *Biotech. Biofuels* **6**; 10.1186/1754-6834-1186-1139 (2013).

3 Cherepanov, P. P. & Wackernagel, W. Gene disruption in *Escherichia coli*: Tc^R and Km^R cassettes with the option of Flp-catalyzed excision of the antibiotic-resistance determinant. *Gene* **158**, 9-14, (1995).

4 Amann, E., Ochs, B. & Abel, K.-J. Tightly regulated *tac* promoter vectors useful for the expression of unfused and fused proteins in *Escherichia coli*. *Gene* **69**, 301-315, (1988).

5 Jobling, M. G. & Holmes, R. K. Construction of vectors with the pl5A replicon, kanamycin resistance, inducible *lac*Zα and pUC18 or pUC19 multiple cloning sites. *Nucleic Acids Res.* **18**, 5315-5316, (1990).
